# Effects on chemotherapy-induced peripheral neuropathy by moderate strength training in combination with whole-body vibration in breast cancer patients

**DOI:** 10.1007/s00520-025-09972-y

**Published:** 2025-10-20

**Authors:** Rebecca Dalferth, Hannah Hebbel, Dirk Bauerschlag, Anne Letsch, Thorsten Schmidt

**Affiliations:** 1 Department of Obstetrics and Gynaecology, University Hospital Schleswig-Holstein (UKSH), Kiel, Germany; 2 Department for Pediatric Pneumology, Allergology and Neonatology, Hannover Medical School, Hannover, Germany; 3 University Hospital Jena, UKJ, Jena, Germany; 4 Department of Internal Medicine II-Haematology, Oncology, University Hospital Schleswig-Holstein (UKSH), Kiel, Germany; 5 University Cancer Centre Schleswig-Holstein (UCCSH), University Hospital Schleswig-Holstein (UKSH), Kiel, Germany

**Keywords:** Chemotherapy-induced peripheral neuropathy (CIPN), Chemotherapy side effects, Resistance training, Breast cancer, Whole-body vibration training (WBV

## Abstract

**Purpose:**

This study aimed to evaluate the effects of a 12-week programme, including moderate strength training with and without whole-body vibration (WBV), on chemotherapy-induced peripheral neuropathy (CIPN) associated symptoms in breast cancer patients undergoing paclitaxel-containing therapy. The goal was to assess the effects on sensory, motor, and autonomic symptoms and on balance, depth sensitivity, and the vibration sensation.

**Methods:**

In this randomized controlled trial, 66 patients were allocated to one of three groups: (i) strength training only (SG), (ii) strength training with WBV (VG), and (iii) a control group (CG) with standard activity advices. Supervised training sessions occurred twice a week during chemotherapy. Outcomes, including balance (Fullerton Advanced Balance Scale, Berg Balance Scale), vibration sensation (tuning fork test), and CIPN symptoms (EORTC QLQ-CIPN20), were assessed at baseline (T0), 6 weeks (T1), and 12 weeks (T2).

**Results:**

No adverse events were recorded, and adherence was high (68.18%). Positive trends indicated stabilized vibration sensation in the SG and VG compared to the control group, but no significant differences were observed. Vibration perception decreased less in the intervention groups, and motor symptoms worsened only in the control group. Balance remained stable across all groups.

**Conclusion:**

Both strength training alone and combined with WBV are feasible and safe during chemotherapy. Although WBV did not provide additional benefits over strength training alone, both interventions showed potential protective effects against CIPN symptoms.

Implications for cancer survivors.

Moderate strength training, with or without WBV, may help prevent or alleviate CIPN symptoms, supporting better physical function during chemotherapy. Early implementation of such programmes could reduce treatment-related side effects.

## Background

Breast cancer is one of the most prevalent cancer worldwide and the most common cancer in women in Germany, with an estimated 74,500 new cases annually [1]. The treatment of breast cancer typically employs a multimodal approach, including surgical procedures, radiotherapy, hormone therapy, and chemotherapy [2, 3]. The role of chemotherapy is particularly important in patients with HER2-positive or triple-negative tumours and in patients with a high KI-67 proliferation index [2].

As chemotherapy can be associated with a variety of side effects, it is essential that the expected benefits of chemotherapy outweigh the potential side effects and long-term damage [2, 3].

Chemotherapy-induced peripheral neuropathy (CIPN) is a common and severe adverse effect of neurotoxic chemotherapeutic agents, occurring most frequently in conjunction with the administration of taxanes, platinum derivatives, and vinca alkaloids [4, 5]*.* Neuropathy symptoms can be classified into five categories, ranging from grade 1 (mild) to grade 5 (death) according to the Common Terminology Criteria of Adverse Events [6]. In patients treated with taxanes, the incidence of developing CIPN ranges from 20 to 50% for grade 1–2, and from 6 to 20% for grade 3–4 [5]. The symptoms of CIPN often disappear once chemotherapy has been completed; however, they can also persist beyond the end of therapy, resulting in long-term effects [5, 7–11].

The defining characteristics of CIPN include sensory, motor, and autonomic nerve disorders, which can present as numbness, paraesthesia, motor limitations, and pain in the hands and feet [2]. These symptoms can be associated with an increased risk of falls, reduced sensory discrimination, and diminished vibration perception[12–15]. Furthermore, these symptoms can have a considerable impact on a patient’s mental well-being and quality of life [10, 16, 17]. The impairments caused by CIPN can persist beyond the end of therapy [7–9].

CIPN represents a potential treatment-limiting factor, insofar as it may necessitate a dose reduction, a delay in treatment, or treatment discontinuation [18–20]. Moreover, it may result in an increased mortality rate [21]. It is anticipated that under-dosing or cycle reduction will result in a loss of therapeutic effectiveness and a poorer outcome [22]. At present, there is a lack of standardized prevention measures and causal treatment approaches for CIPN [5, 23]. Therefore it is important to implement early detection and intervention strategies to prevent the progression of CIPN symptoms [5]. Given the limited number of pharmacological treatments available for CIPN, non-drug therapy modalities are assuming greater significance. As recommended by the German Cancer Society exercise therapy should be used to improve functionality when symptoms of CIPN manifest [5]. Given the limited treatment options for CIPN, it is important to look at studies on the treatment of other causes of PNP, noting that they may have a different pathomechanism.

Physical activity is not contraindicated during and after most oncological treatments [24]. Furthermore, sports interventions in the form of gymnastics and light weight training are suitable for breast cancer patients [25]. Additionally, physical activities are considered safe and feasible for patients with polyneuropathy (PNP) and have been demonstrated to reduce sensory and motor symptoms due to PNP [26]. In particular, balance training has been demonstrated to be an efficacious intervention for PNP [26]. In lymphoma patients, sensorimotor training has been shown to reduce PNP-associated symptoms and enhance the quality of life [27]. If symptoms of CIPN are present, exercise therapy is recommended, including balance exercises, sensorimotor training, coordination training, whole-body vibration (WBV) training, or fine motor skills training [5]. The implementation of low to moderate training intensities, coupled with safety measures such as the use of appropriate footwear and the incorporation of warm-up phases, has been demonstrated to reduce CIPN-associated symptoms and to enhance balance and quality of life [28–31]. The combination of massage, mobilization exercises, physical training, and WBV training has been demonstrated to have a beneficial effect on CIPN-associated symptoms [32]. As stated by Streckmann et al., sensorimotor training and WBV training have great potential for influencing CIPN-associated symptoms [33]. Sensorimotor training has demonstrated considerable promise, as it can have a lasting positive effect on both balance and motor skills [26].

WBV training represents a versatile therapeutic modality. Depending on the individual training goal, the exercises can either be performed statically or dynamically on vibration plates with adjustable frequency and amplitude of the vibration. In addition to vibration plates, vibration dumbbells are another potential option that can also be adjusted in terms of frequency. WBV training has been demonstrated to have beneficial effects on balance in older patients [34, 35] and in individuals with diabetes [36, 37]. In individuals with diabetes and peripheral neuropathy, WBV training has demonstrated to enhance the vibration perception threshold [38]. Additionally, WBV training has been shown to contribute to a reduction in sensory complaints, particularly in the context of pain-associated diabetic polyneuropathy [37, 39, 40]. Streckmann et al. demonstrated that WBV training can be safely performed in CIPN and has the potential to positively influence CIPN-associated symptoms [33].

To date, no studies have investigated moderate strength training with and without WBV as a preventive approach against CIPN specifically in breast cancer patients without preexisting neuropathic symptoms. Existing research has been focused exclusively on patients with established CIPN and has been limited to heterogeneous oncological populations involving various tumour entities, thereby limiting the comparability and transferability of findings to this specific setting. While there are specific training recommendations based on the FITT criteria (F—frequency, I—intensity, T—time, T—type) for managing side effects such as reduced quality of life, fatigue, and lymphoedema, supported by a high level of evidence, no specific training recommendations exist for the primary prevention of polyneuropathies due to insufficient evidence [41, 42]. Therefore, further research is needed to establish a solid evidence base in this area in order to develop targeted exercise recommendations for individuals at risk of developing polyneuropathies.

The primary aim of the present study is to evaluate the impact of moderate strength training with and without whole-body vibration in preventing CIPN-associated symptoms in breast cancer patients undergoing paclitaxel-based chemotherapy. Given the current lack of a clear definition of the relationship between dose and efficacy for WBV, a secondary objective is to determine whether the addition of WBV offers additional benefit to strength training alone. In contrast to previous studies, which initiated exercise interventions after the manifestation of CIPN, this study employs a preventive approach. The intention of early exercise therapy is to reduce the incidence and intensity of CIPN-related symptoms, with the goal of supporting long-term benefits for patients’ daily functioning. The results of this study may help to establish additional WBV training as a component of supportive care for individuals diagnosed with breast cancer.

## Patients and methods

### Study design

This prospective, randomized controlled interventional trial aimed to evaluate the effects of a 12-week resistance training, with or without additional WBV, on CIPN-associated symptoms in breast cancer patients undergoing chemotherapy including paclitaxel. The objective was to analyse the impact of the interventions on clinical and functional aspects of CIPN, including deep sensitivity, balance, vibration sensation and sensory, motor, and autonomic symptoms. These outcome parameters were selected to capture both the clinical manifestations and the functional consequences of CIPN. Sensory deficits such as reduced vibration sensation and deep sensitivity represent typical manifestations of CIPN and can negatively affect balance. Including sensory, motor, and autonomic symptom domains allows an evaluation of the multidimensional neurological and functional impact of CIPN. The interventions were initiated concurrently with the start of paclitaxel chemotherapy, baseline assessments were performed before the first paclitaxel cycle (T0), and the outcome was evaluated at 6 weeks (T1), and 12 weeks (T2). This study adheres to the CONSORT reporting guidelines [43].

### Patient eligibility criteria

The inclusion criteria for this study were as follows: patients with newly diagnosed breast cancer with curative intent and treated with four cycles of dose dense epirubicin/doxorubicin plus cyclophosphamide (EC) over 8 weeks, followed by 12 weeks of paclitaxel mono-chemotherapy. Individuals were excluded if they had an acute infectious disease, severe cardiac disease (e.g., cardiac insufficiency NYHA class III or higher, myocardial infarction > 3 months ago), severe global pulmonary insufficiency, renal insufficiency (GFR < 30%, creatinine > 3 mg/dl), severe neurological disorders, or diabetes mellitus with neuronal involvement (e.g., diabetic neuropathy). Additionally, exclusion criteria included peripheral neuropathy, long-term alcohol consumption with neurological consequences (e.g., Korsakoff’s syndrome), and planned radiotherapy during the intervention.

### Recruitment and randomization

The study participants were recruited between April 2019 and January 2022 at the gynaecological chemotherapy outpatient clinic at the University Hospital Schleswig–Holstein, Campus Kiel, Germany. If a patient expressed interest in participating in the study and met the eligibility criteria, they were provided with comprehensive information and were asked to sign the informed consent form.

Prior to the initial data collection, the participants were randomly assigned to one of three groups in a 1:1:1 ratio, according to the chronological order of their inclusion in the study. The three groups were as follows: the strength training group (intervention group A = SG), the strength and vibration group (intervention group B = VG), and the control group (group C = CG). A statistical calculation was carried out to determine an appropriate sample size.

The sample size was calculated based on previously published data indicating medium to large effects of exercise on CIPN [27]. Participant recruitment encountered significant challenges due to the impact of the SARS-CoV-2 pandemic, which included temporary suspensions and considerable delays. Following a 3-year interim analysis, it was determined that the originally planned sample size could not be achieved due to the ongoing recruitment rate within the study period.

### Interventions

The intervention groups (SG and VG) completed moderate-intensity strength training over the 12-week period of paclitaxel administration. In VG, the strength training was supplemented by additional WBV training. The CG did not receive any additional intervention, but was advised to be physically active on a regular basis in accordance with the S3 guideline for the early detection, diagnosis, treatment, and aftercare of breast cancer [2]. The training sessions were conducted twice weekly for 1 h each in the Department of the University Cancer Center Schleswig–Holstein (UCCSH) at the University Hospital Schleswig–Holstein, Campus Kiel following a strict protocol. The exercises were performed individually under supervision by sports scientists specialized in oncological training. The sport scientists documented the attendance and ensured the exercises were performed correctly. The training programme for intervention groups SG and VG was identical in terms of the warm-up, strength training, and cool-down phases. Following an 8-min warm-up phase on a cycle ergometer, participants performed two sets of 20 repetitions per side on a weight lifting machine. The exercise equipment was from Proxomed®. The machine training regimen involved exercises targeting the latissimus dorsi muscle, the trapezius muscle, the rotator cuff muscles, the quadriceps femoris muscle, and the gluteal muscles. The latissimus dorsi and trapezius muscles were trained using a shoulder blade fixator/support bench; the rotator cuff muscles and the gluteal muscles were trained using a cable pulley; and the quadriceps femoris were trained using a functional bench. The intensity of the strength training tailored to the individual and was set at 60% of the subjective muscle load, based on the average of the last three repetitions of an exercise. To define the individual muscle load participants were asked to rate their exertion on a scale from zero to ten, with zero representing no exertion and ten maximum exertion after performing an exercise, The exertion should be 60% of maximum, which equates to a score of six. If this was not met, the training was adjusted. The starting level of each participant was determined at an initial training session. The training was followed by 4 min of upper body ergometer training and a 6-min cool-down on the upper body ergometer.

The VG was also given a WBV training session, which followed immediately after the warm-up phase. The 10-min WBV training was performed with a vibration plate of the Galileo® Med L type (Novotex Medical GmbH, Pforzheim) and a vibration dumbbell of the Galileo® Mano Med 30 type (Novotex Medical GmbH, Pforzheim). The frequency was increased from 10 to 16 Hz over the course of the study and is shown in Table 1. The amplitude of the side-alternating vibration device was set at 2 mm. The WBV training regimen comprised three 1-min cycles on the vibration plate, with a 1-min intermission between each cycle. The participants were instructed to assume an upright position with their feet positioned at a distance equivalent to the width of their hips and their knees bent at an angle of 25–30°. The hands were positioned around the handrail to provide additional stability. Following the training on the vibration plate, the participants engaged in vibration dumbbell training, alternating sides and following the same set of instructions. The dumbbell was held in the hanging hand with slight flexion of the elbow joint at an angle of 25–30° flexion.

**Table 1:** Progression of the frequency of WBV training

Week	Frequency of the vibration plate	Frequency of the vibration dumbbell
Week 1 to 3	10 Hz	10 Hz
Week 4 to 6	12 Hz	12 Hz
Week 7 to 9	14 Hz	14 Hz
Week 10 to 12	16 Hz	16 Hz

Due to the absence of an established guideline for the optimal configuration of WBV in the context of CIPN prevention and the absence of comparable studies, this study adopted an individualized approach in the configuration of the training. This approach was intended to better align with the specific needs and physical conditions of patients undergoing chemotherapy. The comparability and transferability of training protocols from previous studies are limited by the differences in the patient collective, for example due to a different aetiology of polyneuropathy (for example diabetic polyneuropathy) or a collective with already pre-existing CIPN. When determining the vibration frequency for the present study population, it was of particular importance to ensure a moderate intensity level, with the objective of enabling a combination of resistance and WBV training without overloading the participants.

### Outcome measures

A variety of validated and standardized measuring instruments were employed to investigate the parameters of CIPN-associated symptoms. The primary endpoint was the change in CIPN-associated symptoms, assessed using the EORTC QLQ-CIPN20 questionnaire of the European Organization for Research and Treatment of Cancer for chemotherapy-induced peripheral neuropathy. Secondary endpoints included balance, depth sensitivity, and vibration sensation. The balance was evaluated using the Fullerton Advanced Balance Scale—German version (FAB-D), the Berg Balance Scale (BBS), and the Romberg Test. To assess depth sensitivity and vibration sensation, a neurological tuning fork and the monofilament test were utilized.

The **EORTC QLQ-CIPN20** questionnaire assesses chemotherapy-induced peripheral neuropathy in sensory, motor, and autonomic scales [44]. The validity and reliability of the instrument have been confirmed in studies [45, 46]. The self-assessment included 20 questions, for each of which the patient could choose between four possible answers. The scoring was based on a predefined coding scheme.

The **FAB-D** is a valid and reliable test battery for the assessment of balance ability [47]. The test battery comprises ten standardized tasks. The instructions were first presented by the investigator, then read aloud and evaluated, with a score of 0 to 4 points achievable per task. The maximum score was 40. A score of ≤ 25 signalled an increased risk of falling and a need for action [48]. The **Berg Balance Scale (BBS)** consists of 14 items for balance assessment [49]. These items range from the transition between sitting and standing to more complex motor tasks, such as standing on one leg. The investigator presented the tasks and scored the performance on a scale of 0 to 4, with a maximum of 56 points. A score below 45 indicated an increased risk of falling and an additional need for support in daily life [50]. The **Romberg test** was employed to assess depth sensitivity and included an evaluation of body stability following the closure of the eyes. The participants were positioned with their feet together and arms extended, and were asked to close their eyes. A positive test result, indicative of a disturbance in depth sensitivity, was observed when there was an increase in swaying following the closure of the eyes [51]. A **neurological tuning fork** (c128 Hz/c64 Hz, according to Rydel-Seiffer) was employed to further evaluate depth sensitivity, a rapid method for assessing vibration threshold [52]. The measurements were taken at defined osseous points, including the left and right sides of the distal radius, the first metacarpal, the lateral malleolus, and the first metatarsal. Participants were asked to indicate the point in time at which they no longer felt any vibration. The results were summarized according to upper and lower extremity and differentiated depending on the measurement point. A maximum of eight points could be achieved per measurement point. Higher scores indicated a perception of greater vibration sensation. The **monofilament test** (Semmes–Weinstein test) was conducted using a Neuropen device (Twin-Tip®) to apply a pressure of 10 g, with the objective of evaluating sensory perception. This measuring instrument has the capacity to detect subclinical CIPN [53]. Measurements were taken at the distal phalanges and metacarpophalangeal joints of the fingers and at the distal phalanges and metatarsophalangeal joints of the toes. Participants indicated when they were aware of the pressure applied by the Neuropen with their eyes closed. The results were compared separately for the hands and feet and between measurement times.

This study forms part of the wider Galileo investigation, conducted by the study group ‘Supportive measures: sports and exercise therapy’ from the University Hospital Schleswig–Holstein, Campus Kiel. A subsequent analysis conducted by the same study group examined the effects of the interventions on quality of life, strength development, and fatigue symptoms in this patient cohort.

### Statistical analysis

The statistical analysis was conducted on the sample of participants who had completed at least 70% of the planned training sessions and had taken part in all three measurements, in accordance with the per-protocol analysis approach. The statistical analysis was conducted using Microsoft Excel (version 16.72) and IBM SPSS Statistics (version 29.0.0.0). In Microsoft Excel, the data were recorded in tabular form, processed, and differences in the measured values at the three specified measurement times (T0, T1, and T2) were calculated. Further statistical analysis was carried out using SPSS. The significance level was set at *p* ≤ 0.05, and due to a skewed distribution of the data, a normal distribution was not assumed. The descriptive analysis of the collected data was complemented by the implementation of non-parametric test procedures. The Kruskal–Wallis test was employed to examine the FAB-D, the BBS, the tuning fork test, the monofilament test, and the EORTC QLQ-CIPN20 in order to ascertain any potential differences in the central tendencies of the three independent samples. In the event of statistically significant results, the Mann–Whitney *U* test was utilized to identify and investigate any further group differences. The EORTC QLQ-CIPN20 was analysed in accordance with the provided coding instructions, with a particular focus on the sensory, motor, and autonomic scales. The data from the Romberg test were evaluated by comparing the results of the individual groups.

## Results

### Intervention adherence, safety, and subjects

**Fig. 1:**
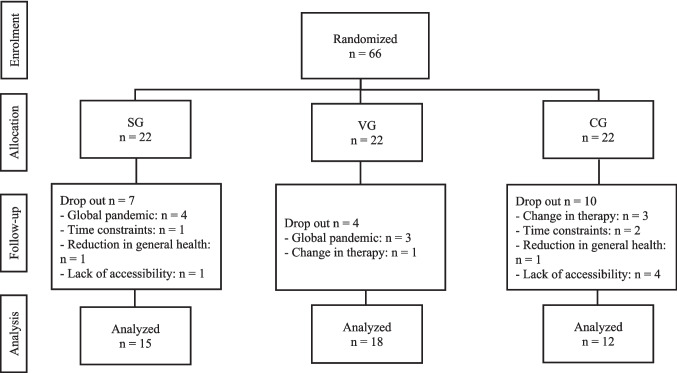
Patient flow and dropout reasons across study groups. Abbreviations: *n* sample size, SG strength group, VG vibration group, CG control group

The study included 66 women aged between 23 and 68 years (mean age: 47.13 years). No statistically significant group differences were identified with regard to age, weight, height, or chemotherapy setting (adjuvant vs. neoadjuvant) (see Table 2). Of the 66 participants, 45 women (68.18%) fully completed the study per protocol. The subjects were distributed among the groups as follows: SG: *n* = 15 (68%), VG: *n* = 18 (82%), and CG: *n* = 12 (55%). The reasons for prematurely ending participation in the study included the implementation of lockdowns and the closure of training rooms during the course of the ongoing global pandemic caused by the SARS-CoV-2 virus (SG: *n* = 4, VG: *n* = 3), a change in the therapy regimen (VG: *n* = 1, CG: *n* = 3), time constraints (SG: *n* = 1, CG: *n* = 2), reduction in general health (SG: *n* = 1, CG: *n* = 1), and a lack of accessibility (SG: *n* = 1, CG: *n* = 4). The patient flow and the reasons for dropout across the study groups are demonstrated in Fig. 1. No adverse events or complications were observed as a result of the study interventions. Recruitment ended with the termination of the research project. An overview of the data from the investigated parameters can be found in Table 3.

**Table 2:** Patient characteristics and chemotherapy setting

**Anthropometric data**	**SG** *n* = 15 (33.33%)	**VG** *n* = 18 (40.00%)	**CG** *n* = 12 (26.67%)	***p* value**
	Mean ± SD	Mean ± SD	Mean ± SD	
Age (years)	47.47 ± 8.82	44.67 ± 7.87	50.42 ± 12.27	0.275
Weight (kg)	69.29 ± 4.54	68.00 ± 0.71	69.00 ± 4.08	0.833
Height (cm)	173.20 ± 1.22	171.84 ± 1.89	171.30 ± 1.06	0.110
**Chemotherapy setting**				**Total**
Adjuvant (%)	26.00	22.00	16.67	22.22
Neoadjuvant (%)	74.00	78.00	83.33	77.78

*n* sample size, *SD* standard deviation, *VG* vibration group, *SG* strength group, *CG* control group. The anthropometric data were recorded at the time of the first data collection. Data source: self-generated

**Table 3:** Results

	**Differences in medians**			**Group comparison with Kruskal–Wallis**
	**Strength group (SG)**	**Vibration group (VG)**	**Control group (CG)**	***p*** ** value**
**Fullerton Advanced Balance Scale**				
T1 − T0	0	0	0	0.140
T2 − T1	0	0	0	0.380
T2 − T0	0	0	0	0.998
**Berg Balance Scale**				
T1 − T0	0	0	0	0.368
T2 − T1	0	0	0	0.066
T2 − T0	0	0	0	0.063
**EORTC QLQ-CIPN20**				
**Sensory scale**				
T1 − T0	7.41	7.41	3.70	0.833
T2 − T1	0.00	1.85	3.70	0.917
T2 − T0	14.81	11.11	7.41	0.611
**Motor scale**				
T1 − T0	0	0	0	0.552
T2 − T1	0	0	2.38	0.853
T2 − T0	0	0	7.14	0.333
**Autonomic scale**				
T1 − T0	0	0	0	0.818
T2 − T1	0	0	0	0.729
T2 − T0	0	0	0	0.883
**Neurological tuning fork**				
**Malleoli**				
T1 − T0	0	− 1.5	− 1	0.904
T2 − T1	1	0	0	0.836
T2 − T0	0	0	− 0.5	0.986
**Ossa metatarsalia**				
T1 − T0	− 1	0	− 0.5	0.815
T2 − T1	1	0	− 0.5	0.761
T2 − T0	0	0	− 1.5	0.979
**Lower extremity**				
T1 − T0	− 2	− 1	− 1	0.894
T2 − T1	1	− 0.5	− 1.5	0.536
T2 − T0	0	− 1	− 2.5	0.981
**Distal radii**				
T1 − T0	1	0	0.5	0.103
T2 − T1	− 1	0	− 1	0.486
T2 − T0	0	0	− 0.5	0.195
**Ossa metacarpalia**				
T1 − T0	1	0	0	0.134
T2 − T1	− 1	0	0	0.517
T2 − T0	0	0	0	0.732
**Upper extremity**				
T1 − T0	2	0	1	0.092
T2 − T1	− 1	0	− 1.5	0.401
T2 − T0	0	0	− 0.5	0.464
**Monofilament test**				
**Feet**				
T1 − T0	0	0	0	0.368
T2 − T1	0	0	0	0.368
T2 − T0	0	0	0	1.00
**Hands**				
T1 − T0	0	0	0	0.619
T2 − T1	0	0	0	0.924
T2 − T0	0	0	0	0.810

*T0* before the first paclitaxel cycle, *T1* 6 weeks later, *T2* 12 weeks later. Lower values in EORTC-QLQCIPN20 indicate less symptoms. Data source: self-generated

### Balance and depth sensitivity

The evaluation of balance ability was conducted using the FAB-D and the BBS. A higher score in both tests is indicative of superior balance ability. The Kruskal–Wallis test was employed to ascertain whether statistically significant differences existed between the groups in the FAB-D and BBS. The results indicated that no such differences were present. Throughout the entire study period, the balance ability of all three groups remained constant, with median differences consistently exhibiting a value of zero. In the BBS all participants achieved scores above 45, indicating an overall high level of balance.

Furthermore, the Romberg test was employed to assess depth sensitivity. No alterations in the test outcomes within or between the groups could be identified over the study period. In summary, the findings indicate that none of the examined groups exhibited notable changes in balance over the study period using these assessment tools.

For an additional measurement of the deep sensory level the monofilament test was used with higher values indicating a higher sensory level. Throughout the study, no changes in sensory level were observed in either the upper or lower extremities in any of the three groups. The median differences remained constant at zero over the entire study period, and no statistically significant differences between the groups were identified at any time point.

### Vibration perception

**Fig. 2:**
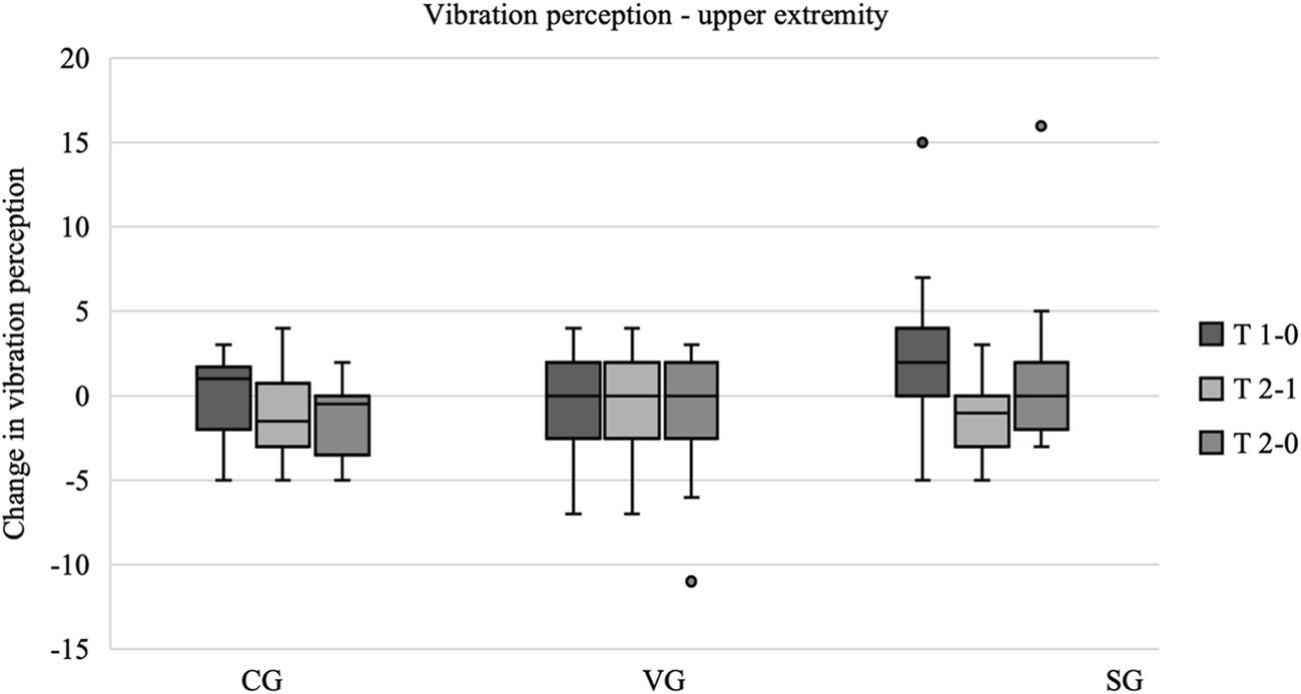
Vibration perception—upper extremity. *The results are presented as boxplots, with the horizontal line representing the median and the dots representing the outliers. The plots show the differences between the measurement times. Positive values correspond to an improvement in vibration perception and negative values to a deterioration. Box and whisker plots of the three groups showing the changes between each data collection point. T 1 − 0 corresponds to the difference in results between the first and second data collection points. T 2 − 1 corresponds to the difference between the second and third data collection. T 2 − 0 corresponds to the difference between the first and third data collection. Median (horizontal line), 95% confidence interval (box), maximum and minimum values (extensions), outliers (°).* Abbreviations: CG control group, VG strength and vibration training group, SG strength training group. Data source: self-generated

The tuning fork test was used to assess vibration perception in the upper and lower extremities. Higher scores were indicative of superior vibration perception. Measurements were taken at bony prominences of the hands (distal radius, first metacarpal) and feet (lateral malleolus, first metatarsal). The results were analysed both for individual measurement points and summarized for upper and lower extremity. With regard to the upper extremity, the vibration sensation remained stable throughout the study period in the intervention groups (median difference T2 − T0 = 0). In the control group, there was an initial improvement (T1 − T0 = 1) followed by a sustained deterioration in vibration sensation (T2 − T0 = − 0.5). A further inspection of the data reveals that the vibration group is the only one that does not deteriorate between T2 and T1. The vibration perception in the upper extremity and the changes over the study period are shown in Fig. 2. A more detailed analysis of the individual measurement points of the upper extremity revealed that the vibration sensation at the ossa metacarpalia remained unaffected between T0 and T2 in all three groups. The control group exhibited a slight decline in vibration sensation at the distal radii (T2 − T0 = − 0.5), whereas no such decrease was observed in the other groups.

**Fig. 3:**
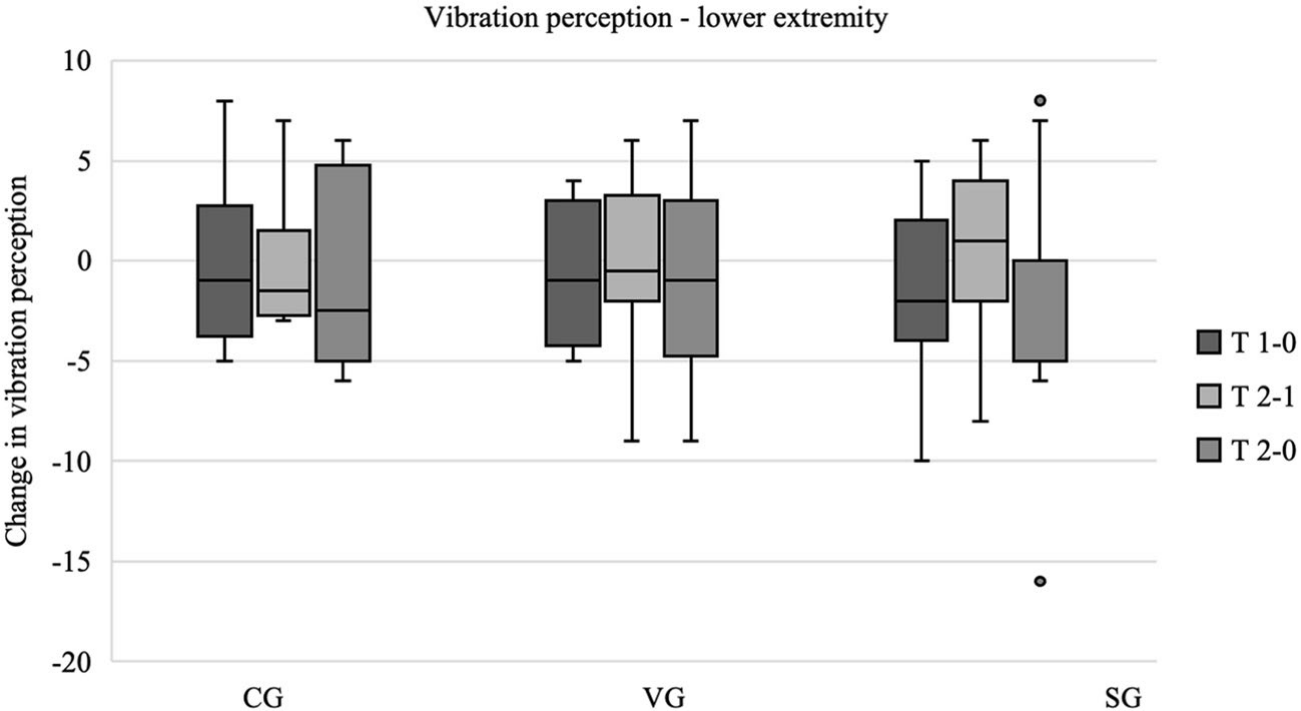
Vibration perception—lower extremity. *The results are presented as boxplots, with the horizontal line representing the median and the dots representing the outliers. The plots show the differences between the measurement times. Positive values correspond to an improvement in vibration perception and negative values to a deterioration. Box and whisker plots of the three groups showing the changes between each data collection point. T 1 − 0 corresponds to the difference in results between the first and second data collection points. T 2 − 1 corresponds to the difference between the second and third data collection. T 2 − 0 corresponds to the difference between the first and third data collection. Median (horizontal line), 95% confidence interval (box), maximum and minimum values (extensions), outliers (°).* Abbreviations: CG control group, VG strength and vibration training group, SG strength training group. Data source: self-generated

In the lower extremity (see Fig. 3), the control group showed a continuous reduction in vibration sensation (median difference T2 − T0 = − 2.5), while the change was less pronounced in the VG (T2 − T0 = − 1). In the SG, vibration perception remained stable over the entire period (T2 − T0 = 0), after an initial worsening (T1 − T0 = − 2) was followed by an improvement (T2 − T1 = 1). Further analyses of the progression of vibration sensation in the lower extremity between T2 and T1 reveal that only the SG demonstrates improvement (T2 − T1 = 1) with the other two groups experiencing a deterioration. Whilst the decline is less pronounced in the VG (T2 − T1 = − 0.5) than in the CG (T2 − T1 = − 1.5). A closer examination of the individual measuring points of the lower extremity revealed that the vibration sensation remained unchanged in the intervention groups, whereas a deterioration was documented in the control group at the ossa metatarsalia (T2 − T0 = − 1.5) and malleoli (T2 − T0 = − 0.5). No statistically significant differences were identified between the groups, either on the upper or lower extremity.

### EORTC QLQ-CIPN20

**Fig. 4:**
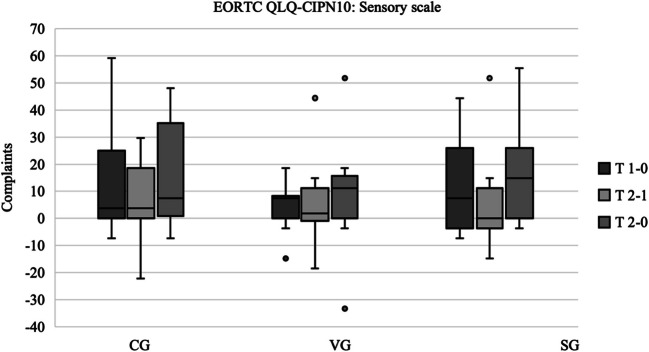
EORTC QLQ-CIPN20—sensory scale. *The results are presented as boxplots, with the horizontal line representing the median and the dots representing the outliers. The plots show the differences between the measurement times. Higher values correspond to more symptoms. Box and whisker plots of the three groups showing the changes between each data collection point. T 1 − 0 corresponds to the difference in results between the first and second data collection points. T 2 − 1 corresponds to the difference between the second and third data collection. T 2 − 0 corresponds to the difference between the first and third data collection. Median (horizontal line), 95% confidence interval (box), maximum and minimum values (extensions), outliers (°).* Abbreviations: CG control group, VG strength and vibration training group, SG strength training group. Data source: self-generated

The evaluation of the Sensory Scale of the EORTC QLQ-CIPN20 demonstrated a notable increase in sensory complaints across all three groups over the course of the study (Fig. 4). This increase was particularly pronounced in the intervention groups (VG: median difference T2 − T0 = 11.11; SG: median difference T2 − T0 = 14.81) compared to the control group (CG: median difference T2 − T0 = 7.41). In the CG, the increase was gradual between the measurement times, whereas in the intervention groups, the greatest increase in complaints occurred between T0 and T1. No statistically significant differences were identified between the three groups.

**Fig. 5:**
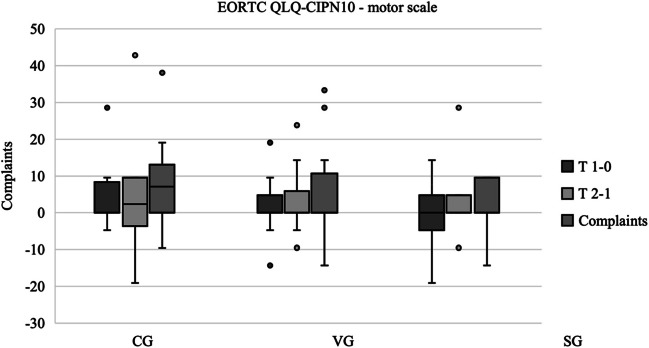
EORTC QLQ-CIPN20—motor scale. *The results are presented as boxplots, with the horizontal line representing the median and the dots representing the outliers. The plots show the differences between the measurement times. Higher values correspond to more symptoms. Box and whisker plots of the three groups showing the changes between each data collection point. T 1 − 0 corresponds to the difference in results between the first and second data collection points. T 2 − 1 corresponds to the difference between the second and third data collection. T 2 − 0 corresponds to the difference between the first and third data collection. Median (horizontal line), 95% confidence interval (box), maximum and minimum values (extensions), outliers (°).* Abbreviations: CG control group, VG strength and vibration training group, SG strength training group. Data source: self-generated

In the evaluation of the motor scale, an increase in motor symptoms over the study period was observed exclusively in the control group (median difference T2 − T0 = 7.14). In contrast, the intervention groups demonstrated no change in complaints (median differences = 0 in each case). The results and changes detected with this questionnaire over the study period with the motor scale are shown in Fig. 5.

With regard to autonomic complaints, no changes were observed in any of the three groups over the study period, with median differences of zero at all time points. No statistically significant differences were identified between the groups for either motor or autonomic complaints.

## Discussion

The German S3 guideline for the treatment and follow-up on breast cancer patients contains recommendations for physical activity in individuals with manifest CIPN [5]. Previous studies have demonstrated that physical activity can positively influence the symptoms of pre-existing CIPN [26–31, 54–57]. The clear beneficial effects observed in these previous studies couldn’t be shown in the present study. However, positive tendencies suggest that the preventive approach may have a positive impact on CIPN-related symptoms. However, the comparability of the present study with previous research is limited, as this study is the first to investigate physical activity as a preventative approach to CIPN-associated symptoms.

Studies from Schönsteiner et al. and Streckmann et al. indicated that WBV can be an efficacious approach with the potential to mitigate the symptoms associated with CIPN [32, 33]. The objective of this study was to initiate a programme of sporting activities at the earliest possible stage, prior to the onset of any symptoms of CIPN in patients undergoing chemotherapy. The preventive use is of particular interest, as it may have the potential to attenuate or even prevent the development of CIPN at an early stage. This is of particular importance given that the established therapeutic options for the treatment of CIPN have thus far demonstrated only limited efficacy [5, 16, 20, 23, 58].

The findings of this study indicate that a sport intervention consisting of moderate strength training and WBV is both feasible and safe during ongoing chemotherapy treatment. Moreover, it provides new insights into this relatively underexplored therapeutic approach with regard to CIPN and the associated symptoms. In the absence of comparable studies, it was necessary to adopt a novel approach to the training design, with a particular focus on the design of the vibration training programme. In order to avoid the risk of overloading, a gradual increase in frequency (10–16 Hz) over a period of weeks was selected in this study. Nevertheless, the selection of this frequency range may be a contributing factor to the lack of significant differences achieved through vibration training. Training at a higher frequency range (18–35 Hz), as conducted in the study by Streckmann et al., may prove an effective approach to increasing the effectiveness of vibration training [33]. It may therefore be advisable to also consider a higher frequency level for the prevention of CIPN.

Although no statistically significant differences were identified between the groups with regard to the examined parameters, positive trends were observed, particularly with respect to the vibration threshold and sensory and motor symptoms. The data collected concerning the vibration threshold demonstrated notable differences between the control and intervention groups. In the upper extremity, vibration sensation remained unaltered in both intervention groups, whereas a decrease was observed in the control group. Vibration sensation in the lower extremity of the intervention groups remained unaltered in the SG and decreased only slightly in the VG. In contrast, there was a major decrease in the CG. The observed decrease in vibration sensation in the control group is a typical and expected side effect of neurotoxic chemotherapy [5, 15]. The positive effects of the interventions on vibration threshold between T2 and T1 are particularly noteworthy given the dose-dependent development of CIPN. These results strongly suggest that the interventions may have a protective effect regarding vibration threshold. It is plausible that through targeted training of the upper extremity with a vibrating dumbbell additional effects may have been achieved in the VG. The objective of the vibrating dumbbell is to facilitate a more precise targeting of the upper extremity.

The assessment of balance ability may have been limited by the use of measuring instruments that may not be suitable for a younger and potentially more active cohort. While the BBS is widely used to assess general balance performance, the FAB-D was included for its higher sensitivity to subtle differences in higher-functioning individuals. The combined use of both scales aimed to provide a more differentiated evaluation of balance capacities. However, it remains possible that even this combined approach lacked the sensitivity required to detect subtle differences between the three groups. Consequently, the detection threshold may have been below the requisite level, potentially obscuring any discrepancies between the groups. This inappropriate detection threshold may be the result of a notable discrepancy between the mean age of the study cohort (median age 47,13 years) and that of the original validation populations for the BBS and FAB-D (median age 73 and 75 years) [47, 49]. At present, no more specific or sensitive test batteries are available for the assessment of balance in a younger and more able-bodied collective. It is therefore recommended that future research projects should aim to develop a test battery that is tailored to a younger and more able-bodied collective. The results of the Romberg test indicated no significant changes. This finding suggests that the test may also have limited sensitivity for a younger cohort without initial neurological impairments.

The sensitivity measured with the 10 g monofilament remained stable in all groups. This may be likewise attributed to the possibility that the 10 g filament lacks sufficient sensitivity to adequately detect minor changes in sensitivity in a young population without initial neurological deficits at the beginning. It may be beneficial to employ monofilaments with a lower pressure in order to facilitate the early detection of polyneuropathic changes in such a collective. Olaiya et al. were able to diagnose subclinical polyneuropathy using a 1 g monofilament [59]. Moreover, Thomson and colleagues established the 6-g monofilament test as the threshold for normal sensory perception in a population with type 2 diabetes mellitus [60]. The early detection of symptoms associated with CIPN is a fundamental step in the prevention of the onset of more severe CIPN [5, 12]. It is therefore recommended that future research should focus on the development of methods capable of detecting subclinical CIPN.

The instruments may have lacked sufficient sensitivity and were complemented by the EORTC QLQ-CIPN20 questionnaire, which was considered to be both valid and reliable [45, 46]. It is therefore of particular importance to consider the questionnaire results carefully. The evaluation of the EORTC QLQ-CIPN20 questionnaire revealed an increase in sensory complaints across all three groups, which aligns with the anticipated side effects of neurotoxic chemotherapy [4, 5]. However, the increase in sensory complaints in the intervention groups was not as continuous as in the CG, and the increase diminished in the second part of the study (T2 − T1). The observed cessation of the increase in symptoms in the strength group is inconsistent with the assumption that the risk of neurotoxicity and the associated CIPN-related symptoms increase with the cumulative dose of chemotherapy [5]. It was observed that the incidence of motor symptoms increased exclusively within the control group. These findings indicate that the administration of sports interventions may be associated with a protective effect on sensory and motor complaints. It should be noted that this effect may only become apparent after a certain period of time, which would require an extended follow-up period. Despite these encouraging trends, no statistically significant differences between the groups could be identified. To better classify the results, a longer observation period and a larger study collective are necessary. Furthermore, future studies should examine whether an earlier start of the training interventions, ideally before the commencement of chemotherapy, can additionally reduce the occurrence of sensory disturbances. With regard to autonomic complaints, no change could be detected across all three groups. This may be due to the fact that autonomic complaints are only rarely a side effect of the chemotherapy used [5].

### Study limitations

A potential limitation of the present study is the inability to blind study participants, trainers, and investigators. This limitation is attributable to the relatively small size of the study team and the obligatory communication between investigators and study participants during the processes of data collection. As a consequence of the lack of blinding of the interventions, there is a possibility that participants who were assigned to the CG by randomization and did not receive additional interventions may have lost interest in completing the study. It is possible that the lack of additional training for participants in the control group was a strong influencing factor, explaining the highest rate of early dropout in this group. The inability to contact the participants in the CG was identified as the most common reason for premature withdrawal from the study in this group (40%). This may indicate a lack of compliance. It is important to note, however, that a significant number of discontinuations observed in the CG were also attributable to modifications in the therapeutic regimen (30%). It is possible that the CG is no longer representative of the original cohort due to the high dropout rate and the fact that only those patients who were potentially healthier completed the study (so-called non-response bias). This may have resulted in an overestimation of the control group. The close observation of patients may also have encouraged them to focus more on their symptoms, which could have led to a potential bias, especially when answering the questionnaires. Another potential limitation of the study is that additional supportive measures, such as nutritional supplementation, local compression, or cryotherapy of the extremities during chemotherapy, were not systematically assessed. Consequently, their potential impact on outcomes could not be evaluated. Furthermore this study did not investigate the underlying mechanisms by which resistance training and WBV may influence the onset and progression of CIPN. Future studies should address this important aspect. The study was also limited by the impact of the global pandemic caused by the SARS-CoV-2 virus. It should be noted that the pandemic restrictions had a significant impact on the recruitment of participants and the realization of the study, resulting in the majority of premature withdrawals from the intervention groups (SG = 57%, VG = 75%). The sample size may have been insufficient, which could explain why no significant differences between the groups could be detected. However, clear trends could be identified in this study that strongly emphasize the relevance of exercise interventions for the prevention and reduction of chemotherapy-associated side effects. Further investigation of these trends in studies with a larger sample size would be beneficial.

## Conclusion

Exercise interventions, particularly strength training combined with WBV, may help prevent CIPN by reducing sensory and motor symptoms, though no additional benefits beyond resistance training alone were observed. This may be due to limited statistical power, suboptimal WBV protocols, or insufficiently sensitive diagnostic tools. Future research should focus on optimizing WBV protocols, refining diagnostic methods, and exploring personalized pre-chemotherapy interventions to improve CIPN prevention strategies.

## Data Availability

The datasets generated during the present study are available from the corresponding author upon reasonable request.
